# Considering the need for movement variability in motor imagery training: implications for sport and rehabilitation

**DOI:** 10.3389/fpsyg.2023.1178632

**Published:** 2023-05-12

**Authors:** Riki Lindsay, Sharna Spittle, Michael Spittle

**Affiliations:** ^1^Institute of Education, Arts and Community, Federation University Australia, Ballarat, VIC, Australia; ^2^Institute for Health and Sport, Victoria University, Melbourne, VIC, Australia; ^3^La Trobe University, Melbourne, VIC, Australia

**Keywords:** motor imagery, motor learning, sport psychology, rehabilitation, neuroscience

## 1. Introduction

Restoring an individual's movement ability when neuromuscular control and motor functions have been diminished is a key challenge facing rehabilitation practitioners. For example, what can practitioners do to reactivate motor function for an individual experiencing upper limb hemiparesis following a stroke when upper limb movement is limited? One potential approach not requiring physical movement is motor imagery training (MIT), the mental simulation of actions in the mind in the absence of overt motor output (Morris et al., 2005; Schack et al., 2014). There is substantial support for MIT in improving key outcomes relevant to rehabilitation settings, such as gait patterns and walking speed in stroke patients (López et al., 2019) and increased muscle activation for individuals recovering from ACL reconstruction (Pastora-Bernal et al., 2021). Of interest to rehabilitation, previous studies have demonstrated that MIT is capable of inducing changes in neural plasticity similar to physical action (Debarnot and Guillot, 2014). For example, Lafleur et al. (2002) reported functional organization in the orbito corticies, rostral portion of the anterior cingulate, and striatum in learning a sequential foot movement task. These findings were similar to those observed in a follow up MIT study, which reported increased cerebral blood flow in the right medial orbitofrontal cortex (Jackson et al., 2003). The potential beneficial effects of MIT may be associated with activation of similar brain regions as motor execution, such as the premotor cortex and fronto-parietal regions (Hétu et al., 2013; Moran and O'Shea, 2020). Alongside neural similarities, MIT may be capable of increasing the excitability of cortical motor representations, with increases in corticospinal excitability reported following MIT (Leung et al., 2013). Interpreted through *reactivation* theory of poststroke motor recovery, such findings have implications in applying MIT for motor recovery. According to *reactivation* theory, increasing the use of affected body parts through motor execution may *reactivate* relevant cortical motor representations and increase excitability, improving sensorimotor function (Murphy and Corbett, 2009). From this perspective, MIT could support the promotion of the neural plasticity and cortical excitability needed to re-establish stable, long-term structural changes in motor networks for motor recovery.

The benefits of MIT for motor learning and re-learning are well established (Lindsay et al., 2021; Simonsmeier et al., 2021). However, previous research indicates that MIT is also beneficial for improving muscle weakness, a significant condition affecting many clinical populations who need rehabilitation (Slimani et al., 2016). Particularly relevant for clinical practitioners is the potential benefits of MIT to reduce negative adaptations in the neuromuscular systems, such as strength loss and muscle atrophy because of injury-related inactivation of the neuromuscular system (Slimani et al., 2016). For example, Paravlic et al. (2019) found that four weeks of MIT combined with physical therapy showed significantly less strength decrease relative to physical therapy alone in patients rehabilitating from a total knee arthroplasty. Additionally, the reduction in strength loss also coincided with significant improvements in chair sit-to-stand performance. Such findings suggest that MIT could be an effective tool for clinical practitioners to reduce strength loss and enhance physical therapy outcomes in clinical settings.

A crucial movement capability is adapting to and navigating the unavoidable variability arising from individual (e.g., height, weight, previous experience) and environmental constraints (e.g., change in walking surfaces) in movement (Davids et al., 2003; Hacques et al., 2021). From an ecological dynamics perspective, variability is functional in ensuring that a movement system (i.e., the individual) remains flexible enough to adapt movement in achieving task demands (e.g., avoiding a pedestrian unexpectedly walking in front of you) (Renshaw et al., 2019; Button et al., 2020). Captured this way, rehabilitation involves developing the capacity to adapt movement to changing environmental demands while satisfying task demands (Button et al., 2020). The issue for the practitioner is how to *safely* expose the individual undergoing recovery to potentially dangerous dynamic contexts, such as avoiding a rock on the ground or stabilizing themselves when an unexpected obstacle emerges in their path.

The simulated nature of MIT may represent a unique solution to safe exposure to dynamic movement contexts in rehabilitation. Grounded in an ecological dynamics perspective, MIT could safely leverage movement variability (e.g., walking on unstable surfaces without assistance) in a way that represents real-world situations, potentially increasing the individual's ability to adapt their movements under changing environments. In an ecological dynamics perspective on motor skill development, the emergence of cognitive states that support motor execution require an individual to be continuously coupled to the environment (Kiverstein and Rietveld, 2018). Although MIT is stimulus-absent, Lindsay et al. (2022a) suggest that MIT can be decoupled from the invariant features of the movement context (i.e., stable, unchanging structure of the movement context) yet coupled to stimulus-sensitive, variant information (e.g., changing light conditions) (Sims, 2020). For example, during MIT, a football player would be decoupled from the action of kicking, but MIT instructions could facilitate coupling to variant information to guide movements by describing features such as crowd noise and changes in muscular forces Lindsay et al. (2022a).

## 2. Ecological dynamics perspective on rehabilitation

Traditional approaches to motor control have viewed variability as noise that is detrimental to the development of accurate and consistent movements (Schmidt et al., 2018). In contrast, an ecological dynamics view proposes that movement emerges from interactions between individual (e.g., physiological composition, body structure), environmental (e.g., different walking surfaces), and task constraints (e.g., the task goal) (Newell, 1986). Thus, movement variability may be functional in allowing adaptation to varying constraints while producing successful outcomes through different joint actions (Chow et al., 2021; Lindsay et al., 2022b). This process is defined as degeneracy, the ability to achieve different movement solutions for the same task, enhancing the capacity of the individual to execute movements under varied constraints (Davids et al., 2003; Button et al., 2020). There are conflicting accounts of the role variability plays in rehabilitation contexts. Some evidence indicates that increased movement variability may be detrimental to key recovery outcomes, such as balance control, with higher center of pressure (COP) variability while standing identified as a key predictor of falls in older adults (Piirtola and Era, 2006). Whereas, further studies suggest that increased COP and center of mass (COM) variability is associated with improved stability for quiet standing in healthy adults (Rajachandrakumar et al., 2018). Despite this conflicting evidence, it is apparent that variability is unavoidable, and it is necessary to develop an individual's ability to adapt their movements appropriately.

Particularly relevant to rehabilitation contexts, research suggests that the capacity to successfully adapt movement is crucial (Caetano et al., 2018; Rajachandrakumar et al., 2018). For example, Caetano et al. (2018) found that older individuals with Parkinson's Disease (PD) demonstrated reduced capacity to adapt gait patterns to unexpected obstacles and targets, culminating in poorer step accuracy. Although these results initially appear to highlight the need to reduce movement variability, an alternative interpretation that aligns with an ecological dynamics approach is that incorporating variability may allow individuals to better deal with changes in environmental constraints (e.g., unexpected obstacles). It is important to note that higher levels of variability do not necessarily equate to improved outcomes. In fact, Cardis et al. (2017) found that to higher movement variability produced larger errors on a bimanual shuffleboard task relative to low variability. These results highlight that movement variability is not akin to increased randomness, rather, it is the purposeful manipulation of relevant task and environmental constraints to facilitate exploration in a way that reveals opportunities for action that appropriately match an individual's movement abilities and characteristics of the environment (Hacques et al., 2021).

## 3. Movement variability and MIT

The ecological dynamics view emphasizes that movement is an emergent process that occurs in a variable, dynamic environment. Subsequently, rehabilitation practitioners should consider accurately representing elements of real-world contexts, such as variability, in rehabilitation programs. A key issue, however, arises with how to safely introduce variability into training. MIT may be a viable solution by presenting simulated variability without exposing individuals to physical risk. Research reviews have highlighted the effectiveness of MIT in movement and rehabilitation contexts, particularly when combined with physical training (Lindsay et al., 2021; Simonsmeier et al., 2021). For example, Lebon et al. (2012) found that 12-sessions of MIT in conjunction with physiotherapy produced greater EMG activation in the quadriceps for individuals rehabilitating from an ACL tear, relative to the control condition. Another concern in rehabilitation is fear of reinjury. Consider a soccer player recovering from an ACL injury sustained during change of direction (COD) during a match, who may experience fear of reinjury and reluctance to engage in COD tasks during rehabilitation (Rodriguez et al., 2019). MIT could be effective in reducing fear of reinjury and increasing confidence of such athletes recovering from ACL reconstructions (Rodriguez et al., 2019). MIT could also help in training COD tasks by successfully generating movement relevant variability (McNeil et al., 2021). Taken together, these findings indicate the potential of MIT to present realistic training environments that replicate environmental variability in a way that facilitates learning and may prepare individuals to successfully engage in physical training by reducing fear of reinjury. The inclusion of variability through MIT may allow training to be representative of real-world contexts without increased re-injury risk.

## 4. Practical considerations for incorporating variability in MIT

Returning to normal activities after injury is a dynamic process, during which practitioners need to consider the reinjury risk of early introduction of complex movements compared to the benefit of exposure to crucial elements of real-world situations to facilitate motor recovery. To optimize patient outcomes, practitioners need to consider which visual perspective will facilitate effective MIT. For MIT focused on strength performance, internal imagery has been found to be effective compared with external imagery (i.e., third-person perspective) (Slimani et al., 2016). Internal imagery involves imaging movements from a first-person perspective or from within their own body. However, practitioners are encouraged to consider the type of skill being trained when selecting visual perspective. For skills that advocate for a specific technique (e.g., gymnastics), external imagery may be more advantageous as it provides more visual information on movement technique (Wright et al., 2022). Given that movements generated using MIT cannot be directly observed, practitioners should consider how they will ensure their patients are engaging in the prescribed MIT. Though the exact content of an individuals' imagery cannot be precisely known, self-report questionnaires such as the Movement Imagery Questionnaire-Revised Second Version (MIQ-RS; Gregg et al., 2010) can provide an indication of an individual's MI ability and their potential engagement in MIT.

Incorporating movement variability into training requires repetition of the movement (e.g., walking) but under conditions requiring varied movement execution to encourage degeneracy (Button et al., 2020). Though this may imply that practitioners should aim to recreate high movement variability in learning environments, practitioners can purposefully manipulate key constraints to leverage appropriate levels of variability based on individual constraints. For example, an individual re-learning to walk after stroke is constrained by reduced neuromuscular control, subsequently walking on uneven surfaces early in the rehabilitation may be overwhelming. A scaffolded approach to MIT could be beneficial to appropriately scale movement variability to the individual.

**Figure 1:**
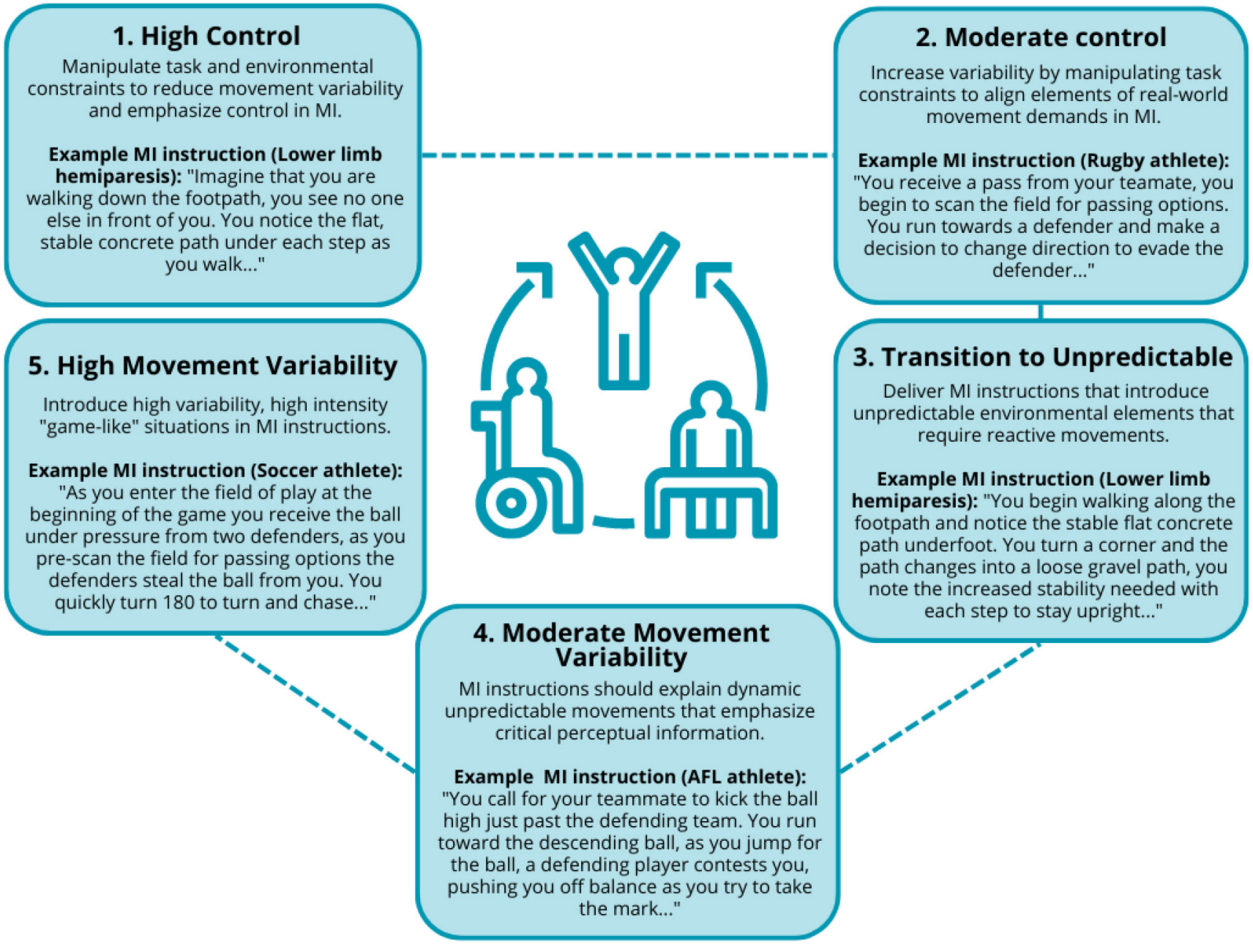
Examples of movement variability incorporated into MIT aligning with the chaos-control continuum (based on Taberner et al., 2019).

Drawing on an ecological dynamics view of motor control and constraints-led approach (CLA) to skill acquisition, Taberner et al. (2019) proposed the chaos-control continuum (CCC) to support the integration of movement variability in the rehabilitation process in sport. The CCC provides a number of key training principles that may guide practitioners on incorporating movement variability into rehabilitation using MIT including: (1) high control—low movement variability activities such as running in straight lines; (2) moderate control— manipulate task constraints to increase variability (e.g., activities that require changing direction with a sport-specific implement); (3) transition to unpredictable/chaotic movements— manipulation of task and environmental constraints to introduce more real-world demands within specified limits (e.g., kicking and receiving on the run without defensive pressure); (4) moderate movement variability—incorporation of dynamic movement situations with increased movement speed and skill execution (e.g., catching and passing under high defensive pressure); and (5) high movement variability—activities reflect task and environmental demands of real-world contexts with no limitations such as walking on an uneven surface with unpredictable pedestrian movements (Taberner et al., 2019). Figure 1 presents practical examples of how MIT instructions can utilize the CCC framework to effectively scaffold movement variability in rehabilitation.

A key benefit of MIT is that it affords rehabilitation practitioners the ability to reintegrate more complex movements with limited risk of reinjury and may allow early activation of motor pathways during periods where movement may be limited or not possible. Subsequently, MIT provides a unique tool to supplement physical training approaches to rehabilitation like the CCC, potentially serving to prime motor pathways and psychologically prepare individuals for more chaotic movement situations and encourage them to explore alternative movement solutions (expressed as movement variability) that match individual motor capabilities (Renshaw et al., 2019). For example, a footballer recovering from an ACL injury may be engaged in phase 1 of the CCC (i.e., high control), during which MIT could be implemented to engage with phase 2 activities, with instructions detailing COD tasks with a football. Alternatively, an individual recovering from lower limb hemiparesis with limited movement may only utilize MIT with low movement variability early in rehabilitation.

## 5. Conclusion

The aim of this paper was to provide some considerations for practitioners in sport and rehabilitation settings incorporating movement variability in MIT for motor skill development. We propose that MI may enable safe engagement with movement variability, potentially supporting adaptation of movement patterns to changing environmental constraints. Informed by a CLA and a CCC framework, MIT may facilitate psychological and neural preparation early in rehabilitation that could contribute to enhanced motor recovery. Practically, the CLA and CCC framework provide key training principles that practitioners can adapt to MIT in moving activities from low variability to high variability and catering to individual constraints in rehabilitation. Practitioners are encouraged to consider MIT as a low-risk strategy for incorporating movement variability into the rehabilitation process and developing adaptable movement skills.

## Author contributions

RL and MS contributed to original conception and structure of this article. RL, SS, and MS contributed to the first draft of the manuscript. All authors were involved in manuscript revision, reading, and approval of the submitted version.
